# Family cohesion and quality of life significantly affecting personality changes in adult epilepsy patients: a case-control study

**DOI:** 10.1186/s12889-024-18861-8

**Published:** 2024-05-23

**Authors:** Jiaai Li, Salamaitiguli Mijiti, Yinyin Xie, Jingqi Lin, Lixia Zhu, Hongmei Meng

**Affiliations:** 1 Department of Neurology, The First Hospital of Jilin University, Changchun, Jilin China; 2 Department of Neurology, The Affiliated Kizilsu Kirghiz Autonomous Prefecture People’s Hospital of Nanjing Medical University, Artux, Xinjiang China; 3 Department of Neurology, The First Affiliated Hospital of Zhengzhou University, Zhengzhou, Henan China

**Keywords:** Epilepsy, Personality, Quality of life, Family cohesion and adaptability

## Abstract

**Objective:**

The goal of epilepsy treatment is not only to control convulsive seizures but also to improve the quality of life of patients. This study aimed to investigate personality changes and the risk factors for their development in adult epilepsy patients.

**Methods:**

A case-control study in a Class III, Class A hospital. The study comprised 206 adult epilepsy patients admitted to the Neurology Department at the First Hospital of Jilin University between October 2019 and December 2021, while the control group consisted of 154 community volunteers matched with the epilepsy group based on age, sex, and education. No additional treatment interventions were determined to be relevant in the context of this study.

**Results:**

There is a significantly higher incidence of personality changes in epilepsy than in the general population, and patients with epilepsy were more likely to become psychoticism, neuroticism, and lie. Epilepsy patient’s employment rate and average quality of life score were significantly lower than that of the general population and had strong family intimacy but poor adaptability in this study. There are many factors affecting personality change: sleep disorders, economic status, quality of life, use of anti-seizure drugs, family cohesion and adaptability. The independent risk factors were quality of life and family cohesion.

## Significance

The study is the first to assess the impact of financial status, family cohesion and adaptability on personality changes in people with epilepsy. It provides a new idea and relevant basis for the prevention and treatment of epilepsy comorbidity.

## Key bullet points

- There is a significantly higher incidence of personality changes in epilepsy than the general population.
- Epilepsy patients were more likely to develop psychosis, exhibit neuroticism, and seek social acceptance by concealing.
- Epilepsy patient’s employment rate and average quality of life score were significantly lower than that of the general population.
- Epilepsy patients enrolled in this study had strong family intimacy but poor adaptability.
- Quality of life and family cohesion were two independent risk factors in epilepsy patients which also were the key to improving personality disorders.

## Background

Epilepsy, the second most common neurological disorder after stroke, not only poses substantial challenges for the sufferers themselves, but the personality changes that often result from this condition lead to a greater burden on the affected individuals’ families and society as a whole. Personality encompasses an individual’s psychological characteristics, including temperament, talent, interests, hobbies, and intelligence, and it is manifested in one’s attitude toward life, individual behaviors, and emotional responses. The development of the bio-psychological-sociological medical model has expanded scholarly interest in better understanding the personality changes that occur in epilepsy patients. Past studies have shown that approximately 50-80% of epilepsy patients experience such changes [1, 2], a significantly higher incidence than that observed within the general population. During seizure intervals, epileptic patients often exhibit personality changes characterized by increased psychoticism and neuroticism scores and decreased extroversion scores [3]. 

Clearly, it is crucial for researchers and practitioners to better understand the nature of these personality changes and their related factors if epilepsy treatment is to advance. However, existing studies exploring these factors remain sparse, with conclusions that vary considerably. For instance, Mao LY et al. identified a relationship between personality and epilepsy course, with extroverted personality found to be independently associated with an epilepsy patient’s educational level. Additionally, they discovered that the course of the disease and education level are both factors affecting the development of personality disorders [3]. In another study by Boldyrev AI et al., a long-term clinical follow-up of 600 epilepsy patients aged 5 to 40 years revealed that social pattern changes, environmental factors, and educational failure have important effects on the emergence of these disorders [4]. 

Due to the gaps in the current literature limiting understanding of these issues, we designed a case-control study to investigate the specific characteristics associated with personality changes in epilepsy patients and the factors that lead to their development. Additionally, we paid special attention to the roles of family cohesion and family adaptability, as these dimensions reflect family health and functionality, which significantly impact the evolution of individual psychological characteristics. The purpose of this study is to increase the understanding of the factors affecting epileptic personality change and provide support for further promoting multidimensional diagnosis and treatment to improve the quality of life of epileptic patients.

## Materials and methods

A total of 206 right-handed patients (age ≥ 16) were employed in this study, all of whom had been admitted to the Neurology Department at the First Hospital of Jilin University between October 2019 and December 2021 and had been diagnosed with epilepsy according to the 2017 International League Against Epilepsy (ILAE) diagnostic criteria. These participants constituted the **epilepsy group** and underwent assessments using Chinese version of the Eysenck Personality Questionnaire (EPQ) (adult, 1984, 88 questions) [5], Quality of Life in Epilepsy Inventory-31 (QOLIE-31, validity: 0.94, reliability: 0.58–0.88) [6], and Family Adaptability and Cohesion Scale II (FACES II) with well validity and reliability [7–9]. Exclusion criteria included left-handedness, serious physical diseases, alcohol dependence, mental disorders, pregnancy, and lactation. A **control group** consisting of 154 community volunteers, matched with the epilepsy group in terms of age, sex, and education, was also included and was likewise tested using the EPQ. Demographic, clinical, antiseizure medication (ASM), economic status, and sleep quality data were collected for all study participants. Statistical analyses were conducted using SPSS25.0 software. The t-test, F-test, chi-square test, correlation analysis, and other statistical methods were employed to evaluate personality traits and related factors. The significance level was set at *P* < 0.05. Prior to multiple linear regression analysis, the independent variables were categorized as follows: family monthly income per capita ≤ 2000 Renminbi (RMB) = 1, 2001–4000 RMB = 2, > 4000 RMB = 3; with sleep disorder = 1, without sleep disorder = 2; with stable job = 1, without stable job = 2; number of antiseizure medication types at the time of study inclusion: one = 1, two = 2, more than 2 = 3; quality of life, family cohesion and adaptability were also treated as variables.

## Results

### Demographic characteristics

The epilepsy group comprised 206 individuals (110 males and 96 females) with a mean age of 34.69 ± 11.77 years. In terms of educational background, 92 participants held a university degree or above, 44 had completed high school or secondary school, and 70 had completed junior high school or a lower level of education. The control group consisted of 154 participants (78 males and 76 females) with a mean age of 35.84 ± 11.27 years. In this group, 75 individuals possessed at least a university degree, 31 had completed high school or secondary technical education, and 48 people had a junior high school education or below. There were no significant differences in gender (*P* = 0.341), age (*P* = 0.351), or education (*P* = 0.746) between the two groups.

### Eysenck personality questionnaire score

The Eysenck Personality Questionnaire (EPQ) consists of 4 subscales, including Psychoticism (EPQ-P), Extraversion (EPQ-E), Neuroticism (EPQ-N), and Lie (EPQ-L), comprising a total of 88 questions. Comparative analysis with the control group revealed significantly higher scores for EPQ-P (*P* = 0.010), EPQ-N (*P* = 0.031), and EPQ-L (*P* = 0.000) among the epilepsy group, with important statistical differences identified (Table 1).

**Table 1:** Personality Traits of epilepsy group and control group

	Epilepsy group(X ± s) *n* = 206	Control group(X ± s) *n* = 154	t	*P*
EPQ-P	44.94 ± 8.77	42.54 ± 8.63	2.589	**0.010**
EPQ-E	50.27 ± 10.11	51.70 ± 11.21	-1.273	0.204
EPQ-N	57.55 ± 12.72	54.61 ± 12.82	2.166	**0.031**
EPQ-L	45.89 ± 10.22	40.79 ± 9.84	4.761	**0.000**

### Factors affecting personality change in epilepsy group

#### 1. Demographic characteristics

Gender, marital status and education level were found to have no significant influence on personality changes.

#### 2. Sleep disorders

Compared to epilepsy patients without sleep disorders, those with sleep disorders were found to have significantly lower EPQ-Extraversion (EPQ-E) scores (*P* = 0.038). This group also showed higher EPQ-Neuroticism (EPQ-N) scores (*P* = 0.007), indicating a more unstable emotional state than patients without sleep disorders. Additionally, individuals in this group are more likely to experience anxiety, tension, irritability, a heightened response to stimuli, and a variety of psychosomatic disorders (Table 2).

#### 3. Economic status

Epilepsy patients with a lower monthly income per capita (*P* = 0.002), unstable job status (*P* = 0.045), and poor quality of life (*P* = 0.001) had higher EPQ-Psychoticism (EPQ-P) scores, indicating a greater likelihood of psychotic traits. Lower monthly income per capita (*P* = 0.001) and poor quality of life (*P* = 0.000) were also associated with higher EPQ-Neuroticism (EPQ-N) scores, suggesting a tendency towards emotional instability. In contrast, patients with a better quality of life had higher EPQ-Extraversion (EPQ-E) scores, indicating a more outgoing personality (*P* = 0.042) (Table 2).

**Table 2:** Sleep disorders and economic status were associated with personality changes

Variable	Sleep Disorders			Economic Status										
				Family monthly income per capita (RMB)				Steady job			Quality of life			
	With (*n* = 58)	Without (*n* = 148)	*P*	≦ 2000 (*n* = 49)	2001–4000 (*n* = 133)	> 4000 (*n* = 24)	*P*	Without (*n* = 131)	With (*n* = 75)	*P*	Poor (*n* = 51)	Medium(*n* = 105)	Good (*n* = 50)	*P*
**EPQ-P**	44.23 ± 7.90	45.22 ± 9.10	0.464	48.77 ± 10.65	43.71 ± 7.60	43.98 ± 8.77	**0.002**	45.87 ± 9.55	43.33 ± 6.99	**0.045**	48.63 ± 11.05	44.35 ± 7.47	42.42 ± 7.60	**0.001**
**EPQ-E**	47.93 ± 9.42	51.18 ± 10.252	**0.038**	48.28 ± 9.82	50.31 ± 10.12	54.12 ± 9.89	0.067	49.84 ± 10.12	51.01 ± 10.12	0.425	49.38 ± 9.24	49.21 ± 10.09	53.39 ± 10.55	**0.042**
**EPQ-N**	61.33 ± 11.73	56.07 ± 12.82	**0.007**	63.27 ± 11.56	55.58 ± 12.21	55.50 ± 14.68	**0.001**	58.23 ± 12.87	56.37 ± 12.44	0.316	64.94 ± 10.56	58.00 ± 11.93	49.07 ± 11.40	**0.000**
**EPQ-L**	46.20 ± 10.153	45.56 ± 10.27	0.785	43.25 ± 11.34	46.73 ± 10.17	46.61 ± 7.02	0.117	46.22 ± 10.77	45.30 ± 9.21	0.538	43.28 ± 10.46	46.35 ± 10.13	47.56 ± 9.84	0.086

**Sleep Disorders** Patients are considered to have sleep disorders if: (1) can not fall asleep half an hour after going to bed or have difficulty falling asleep every night; (2) Less sleep time, shallow sleep at night, easy to wake up; (3) Often wake up 1 h early can fall asleep normally or can not fall asleep normally. **The Quality of life grade** was determined according to the Quality of life in epilepsy Inventory-31 (QOLIE-31) scale score, and the average score of patients in the epilepsy group was 55.12 ± 7.59 points.Poor: < 51.15 (25%), Medium: 51.15–60.40(50%), Good: > 60.40(25%)

#### 4. Clinical features of epilepsy

Age at onset, course of disease, discharge site and discharge laterality based on 16h or 24h EEG, type of seizure and status epilepticus were all determined to have no significant effect on personality changes.(Table 3).

**Table 3:** Clinical features of epilepsy

Characteristic	*n*	%
**Age at onset(n = 206)**		
≦ 18	75	36.4%
19–40	105	51.0%
> 40	26	12.6%
**Course of disease(n = 206) (year)**		
≦ 3	54	26.2%
4–10	74	35.9%
>10	78	37.9%
**Discharge site based on 16h or 24 h EEG (n = 188** ^**#**^ **)**		
Temporal region	78	41.5%
Frontal region	45	23.9%
Frontotemporal region	41	21.8%
Occipital region	2	1.1%
Universality	7	3.7%
Normal	15	8.0%
**Discharge laterality based on 16h or 24 h EEG (n = 188** ^**#**^ **)**		
Focus in the left hemispher	46	24.45%*
Focus in the right hemispher	49	26.05%*
Bilateral	78	41.5%
Normal	15	8.0%
**Type of seizure (n = 188** ^**#**^ **)**		
Focal onset with awareness	5	2.7%
Focal onset with impaired awareness	20	10.6%
Focal to bilateral tonic-clonic	156	83.0%
Generalized onset tonic-clonic	7	3.7%
**Family history of epilepsy (n = 206)**		
With	24	11.7%
Without	182	88.3%
**Status epilepticus (n = 206)**		
With	20	9.7%
Without	186	90.3%

^**#**^18 patients who did not undergo long-term EEG examination were excluded. *All data were rounded to one decimal place. Since the error made the sum of this group 100.1%, the two values with the largest error were rounded down by 0.05% each

#### 5. Antiseizure medication (ASM)

Epilepsy patients who took a higher number of ASMs had higher EPQ-P scores (*P* = 0.001). A subgroup analysis revealed that among 103 patients treated with monotherapy—primarily levetiracetam (*n* = 39) or oxcarbazepine (*n* = 36)—those treated with levetiracetam showed higher EPQ-P (*P* = 0.044) and EPQ-N scores (*P* = 0.021) than those treated with oxcarbazepine. An additional 28 patients were treated with either lamotrigine, topiramate, carbamazepine, or sodium valproate (Table 4).

**Table 4:** Correlation between antiseizure drug treatment and personality changes

Variable	Antiseizure drug treatment						
	Combination of drugs				Drug type		
	Single (*n* = 103)	Two (*n* = 63)	More than 2 (*n* = 40)	*P*	Levetiracetam (*n* = 39)	Oxcarbazepine (*n* = 36)	*P*
**EPQ-P**	43.69 ± 8.37	43.90 ± 8.62	49.43 ± 9.33	**0.001**	45.77 ± 9.0	42.15 ± 5.82	**0.044**
**EPQ-E**	51.18 ± 9.77	49.18 ± 10.46	49.79 ± 10.67	0.461	50.77 ± 9.71	52.41 ± 9.80	0.467
**EPQ-N**	56.03 ± 13.47	59.22 ± 12.03	57.57 ± 12.23	0.309	59.47 ± 12.23	52.40 ± 13.76	**0.021**
**EPQ-L**	46.67 ± 10.17	46.08 ± 9.56	45.03 ± 10.09	0.692	43.99 ± 10.01	47.60 ± 11.41	0.149

### 6. Family cohesion and adaptability

The epilepsy group had family cohesion and adaptability scores of 66.70 ± 11.44 and 45.10 ± 9.35, respectively. Furthermore, the EPQ-P and EPQ-N scales were significantly *negatively* correlated with family cohesion and adaptability, while the EPQ-E and EPQ-L scales were positively correlated with these dimensions (Table 5).

**Table 5:** Correlation between personality changes and family cohesion and adaptability

	EPQ-*P*	EPQ-E	EPQ-*N*	EPQ-L	Family Cohesion	Family Adaptability
**EPQ-P**	1					
**EPQ-E**	-0.086	1				
**EPQ-N**	0.299**	-0.264**	1			
**EPQ-L**	-0.335**	0.087	-0.330**	1		
**Family Cohesion**	-0.398**	0.281**	-0.338**	0.211**	1	
**Family Adaptability**	-0.367**	0.299**	-0.329**	0.160*	0.128*	1

^**^ was significantly correlated with *P* < 0.01 level (bilateral); ^*^ was significantly correlated with *P* < 0.05 level (bilateral)

### 7. Independent influencing factors

The results from the multiple linear regression analysis revealed that quality of life and family cohesion collectively account for 20.3% of the variation in EPQ-P (regression equation: EPQ-*P* = 75.022–0.231 × quality of life − 0.223 × cohesion). Meanwhile, quality of life accounted for 25.1% of the variance rate of EPQ-N (regression equation: EPQ-*N* = 119.96 − 0.582 × quality of life).

## Discussion

Epilepsy is a neurological disorder characterized by a persistent predisposition to seizures, which can result in severe neurobiological, cognitive, psychological, and sociological consequences [10]. The definition proposed by the International League Against Epilepsy (ILAE) and the International Bureau for Epilepsy (IBE) emphasizes the importance of addressing the psychological aspects affecting epilepsy patients in the effective management of the condition. Personality, which encompasses an individual’s physical, social, emotional, and psychological attributes [11], is integral when considering the holistic impact of epilepsy. Normal personality traits typically include some degree of self-awareness and self-control, but epilepsy patients often exhibit traits specific to their condition. Therefore, we designed such a case-control study to elucidate the factors leading to these personality changes while giving special attention to the easily overlooked impact of the quality of life, family cohesion, and adaptability.

Our findings revealed a significantly higher incidence of personality changes in epilepsy patients compared to the general population. Specifically, individuals with epilepsy were more likely to develop psychosis, exhibit neuroticism, and seek social acceptance as measured by concealing themselves than healthy individuals. This aligns with prior research[12, 13] that has also found higher Psychoticism, Neuroticism and Lie scores among epilepsy patients, suggesting that these individuals may be prone to lack of compassion, hostility and aggression, anxiety, irritability, excitement, and a heightened response to stimuli. A tendency to conceal their true feelings to attain acceptance has also been identified, although not all participants had remarkable scores in this dimension as measured by the Lie scale [14]. 

Furthermore, the impact of sleep on brain function and mood has been well-established and is significantly correlated with the onset of mental and psychological disorders. For epilepsy patients, sleep disorders may cause problems related to interpersonal communication, memory, and cognition, leading to declines in reaction speed, visual coordination, attention, and other functions, which can affect patients’ communication and learning abilities. This study also found that epilepsy patients with sleep disorders were more likely to be introverted and emotionally unstable. Therefore, improving sleep quality may present another important approach to limiting personality changes in epilepsy patients.

Epilepsy often requires regular use of anti-seizure medications (ASMs) for its management. While national insurance programs can aid in the management and reimbursement of costs related to chronic diseases, a stable economic foundation is critical for alleviating the financial burden on patients and their families to ensure effective seizure treatment and prevent related complications. In this study, 75 patients (36.41%) reported stable income, but their employment rate was significantly lower than that of the general population [15]. Patients without stable jobs tended to be more aggressive, while those with lower income levels were more likely to exhibit neurotic and psychotic traits.

Moreover, quality of life was also identified as an important factor affecting personality changes. WHO defines quality of life as “an individual’s perception of their position in life in the context of the culture and value system in which they live and in relation to their goals, expectations, standards, and concerns,” [16] providing a concept that can also serve as a primary indicator of one’s overall health. In this study, the average quality of life score among epilepsy patients was 55.12 ± 11.35. Consistent with prior research, this provides additional evidence that epilepsy patients’ quality of life is significantly lower than that of the general population [17]. Furthermore, poor quality of life is also associated with neuroticism and psychoticism. Interestingly, a higher quality of life was found to be correlated with dissembling personality. Hence, helping patients more quickly and effectively adapt to society and providing a foundation for a stable and high-quality life is crucial for the prevention and treatment of personality changes. Family members should as far as possible reduce in front of patients to emphasize the economic burden of the patient’s condition on the family, condition of the patient’s physical condition allows and encourages patients to find a suitable for their work to achieve self-value, the same social members and employers should also be epilepsy patients to give more understanding and tolerance.

Family cohesion, which refers to the emotional connection between family members, is characterized by mutual support, care, harmony, and cooperation. Family adaptability, on the other hand, reflects a family’s ability to adjust to challenges posed by changes in the environment [9]. High family cohesion is associated with close relationships, a harmonious family atmosphere, trust, more frequent and richer emotional communication, and a strong sense of belonging and interdependence. The family functional model theory posits that mutual emotional support within a family contributes to the full realization of a family’s functionality [7]. Therefore, it can be concluded that the quality of family functioning has a significant impact on the development of individual psychological characteristics, which determine the degree of healthy development of physical and mental health characteristics and the formation of healthy personality traits. In this study, the family intimacy score among epilepsy patients was 66.70 ± 11.44, exceeding the national norm of 63.90 ± 8.00, while the adaptability score was 45.10 ± 9.35, which was lower than the national norm of 50.90 ± 6.20. These results indicate that the epilepsy patients enrolled in this study had strong family intimacy but poor adaptability. The findings further revealed that greater family intimacy correlated with a stronger sense of family belonging and interdependence, leading to a lower incidence of psychotic traits. Patients with enhanced family cohesion and adaptability were also more extroverted and more likely to desire social acceptance and were less likely to exhibit neuroticism. It is suggested that patients and family members should maintain high-quality family intimacy and improve patient adaptability under the effective guidance of doctors, communities, and patient associations.

Besides, ASMs are also an important factor affecting the personality change of patients. In this study, epilepsy patients who took more antiseizure medications had higher EPQ-P scores, and patients treated with levetiracetam alone were more likely to develop neuroticism and psychoticism than those treated with oxcarbazepine, which is suggested that clinicians should pay attention to the selection of anti-seizure drugs with little personality change, and pay attention to the prevention and treatment of personality change when adopting the combination medication regimen.

## Conclusion

The goal of epilepsy treatment is not only to control convulsive seizures but also to pay attention to the quality of life of patients, timely detection of changes in patient personality, and give corresponding intervention measures to prevent further deterioration of personality change. This study identified the personality change characteristics and related factors of epilepsy patients. Patients with epilepsy were more likely to become psychoticism, neuroticism, and lie. Sleep disorders, economic status, quality of life, use of anti-seizure drugs, family cohesion and adaptability were the factors that influence personality change in epilepsy. Among them, the independent risk factors were quality of life and family cohesion, which also were the key to improving personality disorders in epilepsy patients.

## Data Availability

The datasets generated and/or analyzed during the current study are not publicly available due [to protect study participant privacy] but are available from the corresponding author on reasonable request.
